# Evaluating the effects of socioeconomic status on stroke and bleeding risk scores and clinical events in patients on oral anticoagulant for new onset atrial fibrillation

**DOI:** 10.1371/journal.pone.0248134

**Published:** 2021-03-18

**Authors:** Kourosh Ravvaz, John A. Weissert, Arshad Jahangir, Christian T. Ruff

**Affiliations:** 1 Advocate Aurora Research Institute, Advocate Aurora Health, Milwaukee, Wisconsin, United States of America; 2 Center for Advanced Atrial Fibrillation Therapies, Aurora Cardiovascular and Thoracic Services, Aurora Sinai/Aurora St. Luke’s Medical Centers, Milwaukee, Wisconsin, United States of America; 3 TIMI Study Group, Brigham and Women’s Hospital, Harvard Medical School, Boston, Massachusetts, United States of America; University of Ioannina School of Medicine, GREECE

## Abstract

**Background:**

The risk of thromboembolism and bleeding before initiation of oral anticoagulant (OAC) in atrial fibrillation patients is estimated by CHA_2_DS_2_-VASc and HAS-BLED scoring system, respectively. Patients’ socioeconomic status (SES) could influence these risks, but its impact on the two risk scores’ predictive performance with respect to clinical events remains unknown. Our objective was to determine if patient SES defined by area deprivation index (ADI), in conjunction with CHA_2_DS_2_-VASc and HAS-BLED scores, could guide oral anticoagulation therapy.

**Methods and findings:**

The study cohort included newly diagnosed patients with AF who were treated with warfarin. The cohort was stratified by the time in therapeutic range of INR (TTR), ADI, CHA_2_DS_2_-VASc, and HAS-BLED risk scores. TTR and ischemic and bleeding events during the first year of therapy were compared across subpopulations. Among 7274 patients, those living in the two most deprived quintiles (ADI ≥60%) had a significantly higher risk of ischemic events and those in the most deprived quintile (ADI≥80%) had a significantly increased risk of bleeding events. ADI significantly improved the predictive performance of CHA_2_DS_2_-VASc but not HAS-BLED risk scores.

**Conclusion:**

ADI can predict increased risk for ischemic and bleeding events in the first year of warfarin therapy in patients with incident AF.

## Introduction

Atrial Fibrillation (AF), the most common heart arrhythmia, affects about 6.1 million American adults, and that number is expected to double over the next 25 years [1,2]. AF increases the risk of stroke approximately 5-fold across all age groups, and strokes incurred by patients with AF are more likely to be fatal or lead to permanent disability [3]. Oral anticoagulation (OAC) therapy effectively reduces stroke risk by more than 60% in patients with AF but at the cost of an increased risk of bleeding, including intracranial hemorrhage [4]. In this light, CHA_2_DS_2_-VASc and HAS-BLED are the two most commonly used risk scores to help determine if OAC therapy will provide an overall net benefit for patients with AF [5].

However, despite the clear benefits of OAC therapy in most high-risk patients with a CHA_2_DS_2_-VASc score of > = 2, OAC therapy remains underutilized. A recent analysis found that as many as 4 in 10 patients with AF who could benefit from OAC are not receiving it due to various concerns, particularly high bleeding risk [6]. Because many of the risk factors for stroke are also risk factors for bleeding, physicians must balance each individual’s risk of stroke and bleeding during their clinical assessment [7]. The difficulty in assessing a patient’s risk profile often leads physicians to overestimate the risk of bleeding and thereby underestimate the benefits of OAC [8,9]. Critically, OACs are withheld disproportionately from patients who gain the most stroke risk reduction. After controlling for common risk factors, AF patients with lower educational attainment, lower-income, and higher age are all less likely to be prescribed OAC [10]. This disproportionately prevents patients with lower socioeconomic status (SES) from receiving the benefits of this important therapy for secondary and primary stroke prevention.

SES is broadly linked to cardiovascular outcomes and could affect the utility of CHA_2_DS_2_-VASc and HAS-BLED score at the point of care, but its impact on anticoagulation adequacy is not fully defined. Indeed, several risk factors included in CHA_2_DS_2_-VASc and HAS-BLED score (e.g., race, tobacco use, and the presence of comorbid diseases, such as diabetes, liver disease, and chronic heart failure) have also been shown to vary across patient SES [11–13]. To better understand the impact of SES on disease processes and outcomes, Area deprivation index (ADI) was developed as a proxy measure for an individual’s SES [14]. ADI is a composite measure of 17 US Census indicators, such as poverty, education, housing and employment, characterized across US census-based neighborhoods. Neighborhood block groups are ordered using the 17 SES variables named above, into a national percentile ranging from 1–100%. Patients living in a neighborhood block group with an ADI of 1% are the least deprived (Highest SES areas). Whereas, patients living in a neighborhood block group of 100% are in the most deprived (Lowest SES areas). Such SES components like income and educational attainment have been linked to poor anticoagulation control with warfarin with low prothrombin (INR) or time in therapeutic range (TTR) [12,13]. It has also been demonstrated that patients with AF living in disadvantaged SES areas have increased morbidity and mortality independent of other risk factors [15]. How ADI impacts the risk profile of patients with respect to currently used risk algorithms such as CHA_2_DS_2_-VASc and HAS-BLED scores to predict clinical events in OAC naïve patients remains poorly defined.

Once it is determined that a patient would likely benefit from OAC therapy, patients and physicians face the difficult decision of selecting either vitamin K antagonists (VKA), such as warfarin, or direct oral anticoagulation agents (DOACs). DOAC therapy has demonstrated similar efficacy to warfarin and is safer with respect to serious bleeding. However, DOACs’ high cost relative to warfarin has raised questions of DOACs’ affordability and cost-effectiveness, especially for patients with TTR >65% who have equivalent outcomes under either warfarin or DOACs [16]. The decision could be particularly fraught for patients with low SES, who are more likely to have risk factors for bleeding on warfarin, such as poor anticoagulation control with low TTR [17]. Patients with low SES and incident AF are therefore in a catch 22. DOACs may be the preferred option considering they are safer with respect to major bleeding. Yet, patients with low SES are unable to afford this option, leaving providers and patients with the choice of either warfarin or no anticoagulant. Therefore, it is necessary to clarify how SES impacts bleeding and stroke risk in patients receiving warfarin therapy for AF.

The objective of this study was to evaluate the impact of SES, measured by ADI, on patients’ risk of stroke and bleeding as determined by commonly used CHA_2_DS_2_-VASc, and HAS-BLED risk scores in newly diagnosed AF patients started on warfarin therapy. We hypothesized that using ADI in conjunction with existing risk scores could enhance anticoagulation therapy risk prediction for AF patients.

## Materials and methods

### Data source

In this retrospective cohort study, we utilized longitudinal electronic health records (EHR) from a large health care network of 15 hospitals, over 150 clinics, and 27 dedicated anticoagulation clinics serving 1.2 million patients each year in eastern Wisconsin and northern Illinois. The inpatient and outpatient EHR utilized in this study included data from anticoagulation clinics, emergency department visits, laboratory results, diagnosis and procedure codes, and medications. Patient-specific EHR data were linked to census tracts and neighborhood-associated ADI using patient addresses. This study was approved by the Aurora Research Subject Protection Program and Institutional Review Board, and informed consent was waived (15-133E).

### Study population

Patients ≥18 years within the health network who initiated warfarin therapy were eligible for inclusion. The first record of warfarin prescription fill was considered the index date, while those patients with only refill warfarin prescription records were excluded. Patients with ≥1 inpatient or two unique (i.e., on different days) outpatient diagnoses of AF in the 12 months preceding warfarin index date were included (S1 Table). Patients were excluded if they had heart valve replacement within the 36 months before index prescription fill date. Additionally, patients with a history of coagulation disorder in the 12 months before index day were excluded. Any record of VKA or DOAC within 12 months before AF diagnosis or with DOAC prescriptions overlapping within 60 days prior to index day were removed. Finally, patients with ≥5 INR checks following index day were included.

### Outcome calculation

The study period for each patient began 29 days following the index day. The first 28 days of therapy post-index date were excluded to allow for the warfarin dose titration, a period characterized by increased INR lability. We defined TTR as an INR between 2.0 and 3.0, as measured by Rosendaal linear interpolation [18]. TTR was calculated using a previously validated algorithm that defines the outpatient warfarin exposure period using a combination of prescription records and INR checks [19]. This method for calculating warfarin exposure and TTR was validated by chart review and demonstrated high predictive performance (κ = 0.84) [20].

Clinical outcome metrics included bleeding and ischemic events. Bleeding events included a composite of intracranial hemorrhage or hemorrhagic stroke, gastrointestinal (GI) hemorrhage, or other bleeding. Ischemic events included a composite of ischemic stroke, transient ischemic attack (TIA), or systemic embolic events. Validated ICD-9 coding algorithms were used to identify outcome events based on published studies (S2 Table). ICD-9 codes were then translated to ICD-10 using a conversion tool [21]. Patients were followed from index day until the earliest incidence of one of the following events: VKA discontinuation, DOAC prescription (i.e., apixaban, dabigatran, edoxaban, rivaroxaban), death, 12 months post-index day, a bleeding event, or an ischemic event.

### Baseline characteristics/Covariates

Patient demographic and behavioral characteristics were recorded on the index day. Comorbid conditions included in CHA_2_DS_2_-VASc and HAS-BLED calculations were identified in the 12-months preceding index day using ICD-9 and ICD-10 codes in the outpatient and inpatient EHR based on previous literature [22–24]. A complete list of interacting medications is provided in the Supporting information. Patient address at index day was linked to census track, which was associated with the national ADI scores as calculated and described in Kind *et al* [25]. Patient ADIs are provided in national percentile rankings at the neighborhood block group level from 1 to 100, with l indicating the least disadvantaged and 100 as the most disadvantaged neighborhoods nationwide.

### Statistical analysis

Differences in TTR between ADI quintiles were assessed using one-way ANOVA. A *p*-value of <0.05 was used as the cutoff for a significant difference in outcomes. Multivariate analysis for predicting ischemic and bleeding events was calculated using the cox proportional hazard model. Survival analysis was conducted using Kaplan-Meier for ischemic and bleeding events, stratified by Age as well as national ADI.

Kaplan-Meier curves for both bleeding and ischemic events were evaluated across ADI quintiles defined with equal width (cut points of 20, 40, 60, and 80%) as is commonly done [26]. To evaluate age in combination with ADI values, ADI quintiles were grouped into a single cut-point according to the risk of ischemic and bleeding events as determined by log-rank test of Kaplan-Meier curves of each ADI quintile.

Additionally, Concordance index (C-index) was also calculated for high- and low-risk cohorts as determined by CHA_2_DS_2_-VASc and HAS-BLED with and with ADI quintiles.

Analyses were conducted in R 3.2.3 [27].

## Results

A total study population of 7274 met all eligibility criteria. Across demographic characteristics, the study cohort was predominately white, aged >60 years old and male (Table 1).

**Table 1:** Characteristics of the study cohort.

*Definitions*	*N (%)*	*Definitions*	*N (%)*
Total Number of Patients	7274 (100%)	Medical history: Two or more of the below comorbidities	4333 (60%)
Sex (female)	3342 (46%)	Hypertension	3796 (52%)
Age (<60 years)	733 (10%)	Diabetes	1764 (24%)
Age (<65 years)	1337 (18%)	Peripheral arterial disease	837 (12%)
Age (65–74 years)	2021 (28%)	Chronic heart failure	1747 (24%)
Age (>75 years)	3916 (54%)	Stroke	612 (8%)
Interacting medications^a^	2792 (38%)	Pulmonary disease	571 (8%)
Medication predisposing to bleeding	1954 (27%)	Hepatic disease	3633 (50%)
Tobacco use (within 2 years)	756 (10%)	Renal	1066 (15%)
Alcohol use	115 (2%)	Coronary artery disease/Myocardial infarction	2135 (29%)
Race (non-Caucasian)	379 (5%)	History of bleeding	897 (12%)

^a^An interacting medication is defined as any medication that interacts with warfarin. A complete list of medications included is provided in Supporting information.

The average national ADI of the population was 45.85 (SD = 19.64). Patients in the first ADI quintile (ADI <20%) had a significantly greater number of low stroke risk patients as measured by CHA_2_DS_2_-VASc, compared with the remaining 4 lower ADI quintiles. However, the distribution of high- and low- bleeding risk patients, as measured by HAS-BLED, did not significantly differ between ADI quintiles (Table 2).

**Table 2:** The scoring schemas stratified by ADI.

	ADI Quintile					
	1 ADI <20%)	2 (ADI 20–39%)	3 (ADI 40–59%)	4 (ADI 60–79%)	5 (ADI ≥80%)	p-Value
*Risk score*	*N (%)*	*N (%)*	*N (%)*	*N (%)*	*N (%)*	
CHA_2_DS_2_-VASc Low-risk	104 (19%)	396 (15%)	308 (12%)	141 (12%)	57 (13%)	<0.01
HAS-BLED Low-risk	336 (62%)	1415 (55%)	1366 (53%)	633 (56%)	247 (54%)	0.10
Total Number of Patients	543 (7%)	2581 (36%)	2555 (35%)	1139 (16%)	456 (6%)	

### Time in therapeutic range

Across the entire population, the mean TTR was 58% (SD = 25 and the median TTR was 61% (S1 Fig). TTR varied significantly between ADI quintiles. The study cohort had an average of 17.5 INR checks (SD = 8.3), with more than 85% of the population having ≥10 INR checks (after 28 days stabilization) during the first year of therapy. The average follow-up was 339 days. TTR varied significantly by ADI (Table 3). Several INR- and TTR-based metrics used as proxy measures for OAC therapy quality were significantly worse for patients living in higher deprivation neighborhoods than counterparts living in lower deprivation areas. However, Patients residing in the highest deprivation areas did have a significantly higher number of INR checks.

**Table 3:** TTR and clinical event outcomes stratified by ADI quintiles.

	1 (ADI <20%)	2 (ADI 20–39%)	3 (ADI 40–59%)	4 (ADI 60–79%)	5 (ADI ≥80%)	p-Value
Mean TTR (SE)	57.6% (55.4–59.8)	57.9% (56.9–58.8)	58.7% (57.8–59.7)	55.8% (54.4–57.2)	53.7% (51.2–56.1)	<0.05
Percent of cohort with TTR >65%	44.75%	43.74%	45.60%	40.56%	37.50%	<0.05
Percent of cohort with TTR <40%	27.26%	27.12%	26.38%	30.29%	32.68%	<0.05
INR checks (SE)	17.0 (16.4–17.7)	17.3 (17.0–17.6)	17.6 (17.2–17.9)	17.6 (17.1–18.1)	18.8 (17.9–19.7)	<0.05
Thromboembolic Events N (% incidence)	17 (3.13%)	57 (2.21%)	67 (2.62%)	39 (3.42%)	17 (3.73%)	0.12
Major Bleeding Events N (% incidence)	18 (3.31%)	132 (5.11%)	128 (5.01%)	42 (4.13%)	36 (7.89%)	0.02
Death N (% incidence)	21 (3.87%)	95 (3.58%)	101 (3.95%)	55 (4.83%)	17 (3.73%)	0.58

Additional sensitivity analysis for TTR at 8 weeks following warfarin initiation and 26 weeks following initiation demonstrate demonstrates that TTR worsened over time for the two most deprived quintiles and improved over time for the three less deprived quintiles (S1 Fig).

### Clinical outcomes

Out of the entire cohort, 7.67% of patients experienced a bleeding or ischemic event, representing 558 total events. Patients were nearly twice more likely to experience a bleeding event than an ischemic one (4.97% vs. 2.71% of the cohort, respectively). Bleeding events were defined as GI (n = 198), intracranial (n = 29), or other major bleeding (n = 134). Ischemic events were defined as stroke-TIA (n = 182) or systemic embolic events (n = 15). Mortality (289 deaths) occurred in 3.97% of the cohort. A total of 308 (4%) patients switched from warfarin to DOAC during the first year of therapy.

When the effect of ADI on ischemic events was evaluated, patients living in the two highest quintiles (ADI ≥ 60%) experienced higher efficacy events compared to patients living in lower deprivation areas that met statistical significance in the elderly (>75 years).

**Fig 1:**
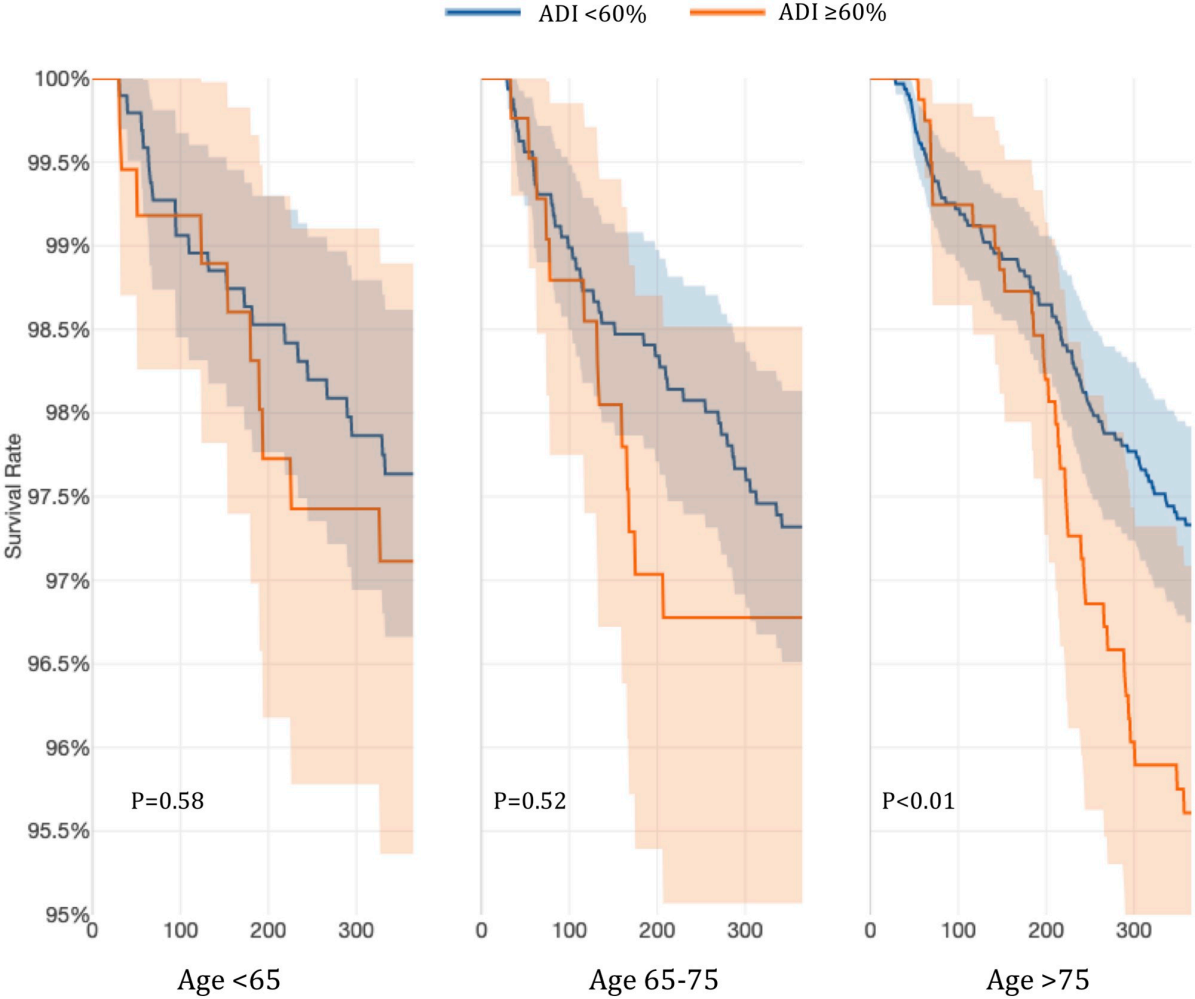
Probability of thromboembolic ischemic events stratified by age and ADI ≥60%.

When age groups used in CHA_2_DS_2_-VASc, as well as ADI quintiles, were used to stratify the cohort, we found that older patients >75 years of age living in the low SES areas had a significantly higher risk of efficacy events (Fig 1).

Additionally, when ADI was added to CHA_2_DS_2_-VASc score, the c-index increased beyond the bounds of the standard error from 0.54 (SE = 0.01) to 0.57 (SE = 0.02), indicating that ADI quintile plus CHA_2_DS_2_-VASc increased the performance to predict stroke and TIA.

**Fig 2:**
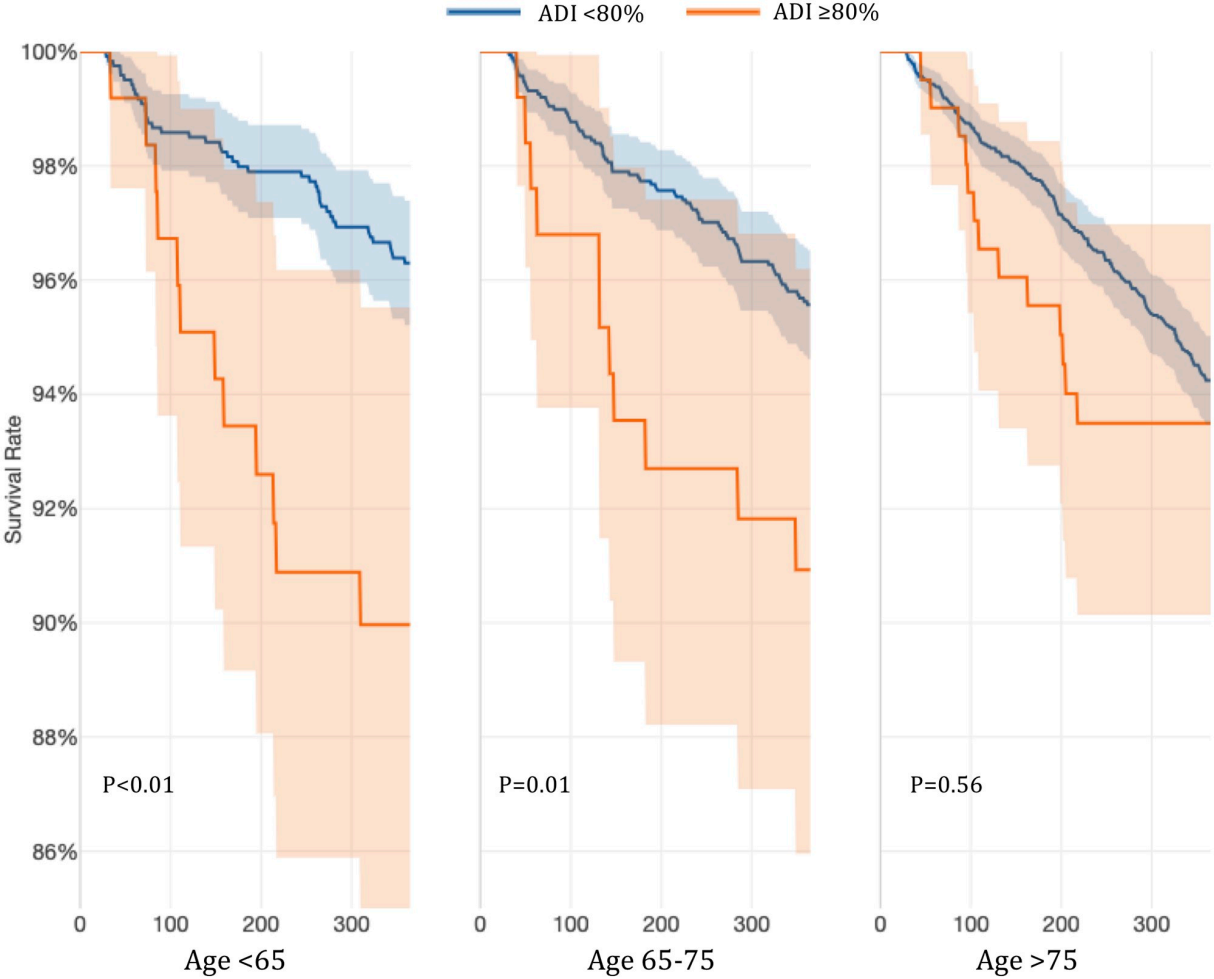
Probability of safety events stratified by age and ADI ≥80%.

In evaluating bleeding events, patients living in very low SES areas, defined as ADI≥80%, had a significantly higher risk of bleeding episodes. When we stratified the population by both age group and ADI ≥80%, we found that younger patients were more likely to experience bleeding events in the ADI ≥80% group while elderly (>75 years) were at high risk regardless of ADI (Fig 2).

When ADI quintiles were added to the HAS-BLED score, the c-index increased from 0.64 (SE = 0.01) to 0.65 (SE = 0.02), but the increase was not greater than the standard error.

**Fig 3:**
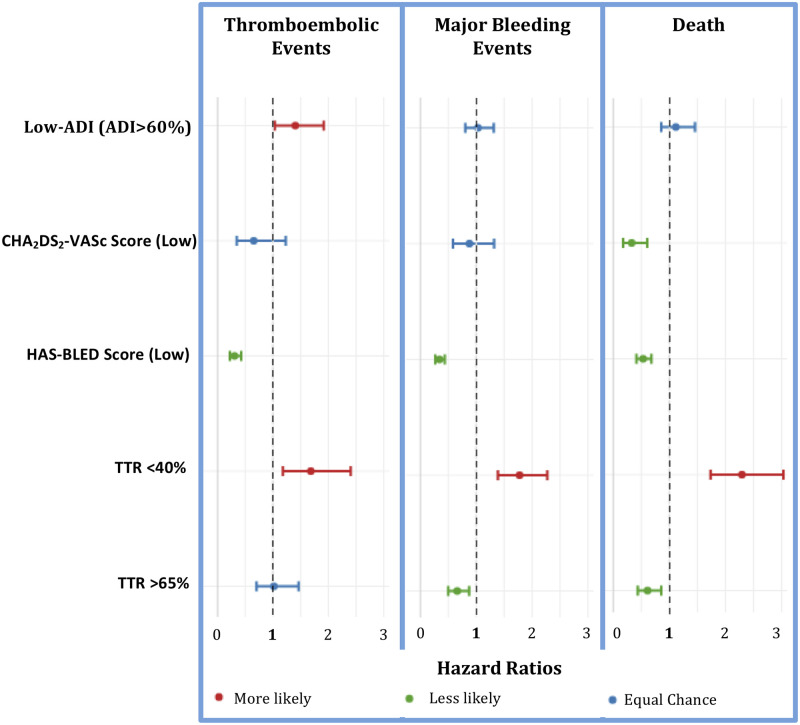
Multivariate cox regression analysis to predict clinical events with Low-ADI (ADI>60%), CHA_2_DS_2_-VASc score (Low), HAS-BLED score (Low), TTR<40%, and TTR>65%. Patients with TTR <40% are at significantly higher risk for stroke-TIA, all bleeding events and death, while patients with a low-risk HAS-BLED score are at significantly lower risk for stroke-TIA, all bleeding events and death. Patients living in more deprived neighborhoods of ADI >60% were at higher risk for ischemic events, but not death or bleeding events. S3 Table demonstrates multivariate analysis using cox regression to evaluate the risk of ischemic, bleeding and death in patients using warfarin as secondary stroke prevention (n = 612). Patients with a TTR <40% were significantly more likely to suffer from ischemic events and death.

Multivariate analysis using cox regression to evaluate the impact of ADI-quintiles, scoring schemas and TTR<40% or TTR>65% during the first year on clinical outcomes is presented in Fig 3.

## Discussion

In this retrospective cohort of OAC-naïve patients with newly diagnosed AF who were started on warfarin, a higher ADI significantly predicted poor anticoagulation control with low TTR values as well as increased risk of clinical efficacy and safety events. For patients living in low SES areas, ADI≥60% predicted thromboembolic efficacy events, and for patients living in very low SES represented by ADI ≥80% predicted thromboembolic efficacy, as well as bleeding events for patients with AF on warfarin therapy, even after controlling for common risk factors. Patients >75 years of age and living in low SES are particularly at risk for efficacy or thromboembolic ischemic events. While, patients younger than 75 years old living in very low SES are at higher risk for bleeding events. It is also noteworthy that patients older than 75 years of age are at greater risk for bleeding events, regardless of ADI.

Patients considered high-risk by either CHA_2_DS_2_-VASc or HAS-BLED did not significantly differ between ADI quintiles, except for a slightly greater number of low-risk patients present in the lowest ADI quintile (High SES). Yet, the performance of CHA_2_DS_2_-VASc scoring schema to predict ischemic events within the first year could be improved with ADI stratification. These differences may become more prominent if extended over a longer period and require further investigation. This suggests that using ADI to stratify patients when calculating a CHA_2_DS_2_-VASc score could improve clinical event prediction for patients prescribed warfarin. This is also demonstrated in the multivariate analysis where ADI is significantly predictive of stroke-TIA even when TTR and CHA_2_DS_2_-VASc score is included in the analysis.

In contrast, although the discrimination for HAS-BLED trended higher when the ADI quintile was included, it did not result in a significant difference during one-year follow-up. It is worth considering that patients living in very low SES areas defined as ADI≥80% were underrepresented in this cohort. Although 20% of all US residents live in very low SES areas, only 5% of our cohort were in very low SES areas. It is unlikely that patients living in very low SES areas have a lower incidence of AF, as has been demonstrated for Southern and Mountain West states that have a greater proportion of patients with ADI ≥80% [28]. Therefore, it is possible that this cohort is underrepresented in our study because patients in very low SES areas are less likely to receive anticoagulation therapy.

It is possible that utilizing ADI to stratify patients in areas with a higher proportion of patients in a very low socioeconomic group would have an even greater impact on predictive performance.

These results highlight some of the issues that clinicians face when choosing anticoagulation for stroke prophylaxis in patients living in low SES areas. Older patients living in low SES areas are more likely to have a stroke while on warfarin, while younger patients living in very-low SES areas are more likely to have bleeding events on warfarin. The challenges of warfarin therapy are further demonstrated with TTR. Patients living in very low SES areas are more likely to have TTR<40%, less likely to have TTR>65%, and have a significantly lower average TTR than patients living in higher SES areas.

Thus, regardless of the patients age, patients of any age are more likely to suffer from either a stroke or bleed event if living in a low SES area on warfarin–although the results are more nuanced when broken down by age group between bleeding and efficacy events.

Patients living in the two highest ADI quintiles have no significant differences in their CHA_2_DS_2_-VASc scores or HAS-BLED scores compared to the second and third quintiles. This suggests that the burden of known risk factors included in these two scores are comparable between ADI quintiles, and that the additional risk of ischemic and bleeding events in the higher ADI quintiles is related to risk factors beyond those included in the CHA_2_DS_2_-VASc and HAS-BLED. The increased risk of stroke in older patients living in high ADI areas demonstrates that these patients are particularly in need of additional stroke preventative measures, both careful INR monitoring and perhaps additional compliance, educations, or non-pharmacological interventions to impact modifiable behaviors such as diet, exercise, tobacco cessation or weight loss might be needed. Additional studies are warranted to identify and rectify factors underlying the risk of ischemic and/or bleeding events for patients living in low and very low SES areas and determine the benefit of warfarin over alternative therapies which can impact modifiable risk factors for stroke and bleeding.

Several observational studies have demonstrated effectiveness of such stroke reduction methods such as diet and exercise and obesity [29,30]. For younger patients <75 years old in low SES areas who do not seem to exhibit an increased risk of stroke, their bleeding risk is significantly increased. This indicates that warfarin should be very carefully managed for these patients as well, with a need to carefully monitor high INRs and ensure that modifiable bleeding risk factors such as diet and alcohol consumption that increases the risk of bleeding are better controlled.

Patients in very low SES areas required a significantly higher number of INR checks than patients in higher SES areas. Reduced compliance in OAC therapy over time, reduced TTR over time, among other possible explanations, could provide insight into why the risk of ischemic and bleeding events among patients living in more deprived neighborhoods seems to diverge around 180 days into warfarin therapy (S1 Fig). This would suggest that patients living in very low SES areas need even more INR checks, especially in beyond 180 days post warfarin initiation, additional compliance counseling, and educational interventions to increase their TTR and reduce stroke and bleeding risk. However, these interventions could place an additional logistical and financial burden on these patients potentially already under significant financial obligation. An alternative could be the prescription of DOAC therapy, considered the preferred choice over warfarin, which does not require frequent anticoagulation monitoring or dosage adjustments. However, for high-ADI patients, this might not be a viable option due to higher financial burden and out-of-pocket expenses. Further, non-pharmaceutical interventions such as left atrial appendage occlusion, the impact of regular exercise regimes, improved diet, and weight reduction to reduce AF burden on adverse outcomes in patients living in low SES areas needs to be assessed.

These results demonstrate that warfarin therapy alone is insufficient stroke prophylaxis for patients living in low SES areas and needs to be supplemented by closer monitoring, improved compliance and rectification of other modifiable factors to ensure appropriate risk profile improvement and therapeutic efficacy.

Recently, two additional risk scores, the PROSPER score and the Geisinger model, have been developed with a similar goal of assessing the pretreatment risk of low-quality VKA therapy [13,31]. However, PROSPER and Geisinger’s ability to predict clinical events has not been studied, and consequently, were not used in this study. A fourth risk score, the TIMI-AF score, was recently developed to identify patients with AF who are at increased risk for clinical events from warfarin versus edoxaban therapy [32]. TIMI-AF score reported a slightly higher C-index (0.68) for predicting clinical events. However, similar to PROSPER and Geisinger models, TIMI-AF has not yet been evaluated in a prospective trial setting alone or with ADI.

## Limitations

This study is based on a retrospective identification of clinical events using a combination of ICD-9 and ICD-10 codes within EHR data. Although ICD-9 codes have been previously validated, the specific clinical validity of their ICD-10 translations remains unknown. We also did not distinguish between patients who experience paroxysmal versus persistent AF that is correlated with stroke risk and OAC therapy quality. Additionally, although we captured all INR values and ICD-codes in the healthcare network, it is possible that patients who sought out-of-network care were not captured. We also acknowledge that there are several variables beyond those included in CHA_2_DS_2_-VASc or HAS-BLED such as smoking, renal failure, BMI or blood pressure control have impacts on stroke and bleeding events and are therefore not considered in this analysis.

## Conclusion

The predictive performance of CHA_2_DS_2_-VASc but not HAS-BLED, was significantly impacted by ADI. Living in higher deprivation areas is predictive of increased risk for ischemic events and living in very high ADI ≥80% also increased the risk for bleeding events in incident AF patients prescribed warfarin in the first year after AF diagnosis. Additionally, ADI was predictive of worse TTR outcomes, including lower mean TTR, and lower probability of achieving TTR >65%. These results suggest alternative anticoagulation therapy options such as DOACs should be considered for these patients. Additional prospective studies are needed to determine if these patients preferentially benefit from alternative treatment with DOACs.

## Supporting information

S1 Fig Average time in therapeutic range (TTR) within each ADI quintile at weeks 8, 26, and 52 of warfarin therapy. Error bars indicate the 95% confidence interval.*indicate that there are significant differences in average TTR between the ADI quintiles at that point in time (p-value<0.05).
(DOCX)

S1 Table List of ICD-9 codes used for cohort identification and to identify comorbidities. (DOCX)

S2 Table List of ICD-9 and ICD-10 codes used to identify clinical events. ^a^All clinical events were identified using inpatient diagnosis only.
(DOCX)

S3 Table Multivariate analysis using cox regression to predict ischemic and bleeding events in patients with a previous history of stroke prior to initiating warfarin (secondary stroke prevention). Variables included ADI with a threshold of 60%, as well as TTR thresholds of 40% and 65% in the first year of therapy. Hazard ratios in bold indicate statistical significance (p<0.05). Ischemic events include Systemic Embolic Events and Stroke TIA. Bleeding events includes, GI-bleeding, non-GI bleeding and intracranial hemorrhage.
(DOCX)
