# Supplementary material for: Evaluating the effects of socioeconomic status on stroke and bleeding risk scores and clinical events in patients on oral anticoagulant for new onset atrial fibrillation
Source: PLoS One. 2021 Mar 18;16(3):e0248134. doi: 10.1371/journal.pone.0248134 (PMC7971564; doi:10.1371/journal.pone.0248134)
Supplement: S1 Fig — Error bars indicate the 95% confidence interval.*indicate that there are significant differences in average TTR between the ADI quintiles at that point in time (p-value<0.05). (DOCX) [file pone.0248134.s001.docx]

S1 Fig. Average Time in Therapeutic Range (TTR) within each ADI Quintile at weeks 8, 26, and 52 of warfarin therapy. Error bars indicate the 95% confidence interval.*indicate that there are significant differences in average TTR between the ADI quintiles at that point in time (p-value<0.05)
