# Supplementary material for: Evaluating the effects of socioeconomic status on stroke and bleeding risk scores and clinical events in patients on oral anticoagulant for new onset atrial fibrillation
Source: PLoS One. 2021 Mar 18;16(3):e0248134. doi: 10.1371/journal.pone.0248134 (PMC7971564; doi:10.1371/journal.pone.0248134)
Supplement: S1 Table — (DOCX) [file pone.0248134.s002.docx]

# **Supporting information**

| Table S1. List of ICD-9 codes used for cohort identification and to identify comorbidities | | |
| --- | --- | --- |
| Comorbidity | ICD-9 Codes | Comment |
| Atrial Fibrillation | 427.31, 427.32 | One inpatient or two outpatient diagnoses, on different days. Consistent with retrospective evaluation of warfarin and dabigatran in three distinct evaluations (1,2,3) |
| Valvular Disease | 35.05, 35.06, 35.07, 35.08, 35.09, 35.2  V42.2, V43.3 | One inpatient or two outpatient diagnoses, on different days. Consistent with retrospective evaluation of warfarin and dabigatran (2,4) |
| Coagulation Disorder | 269.0, 286.0-286.8, 286.52, 286.53, 2886.59, 289.91 | One inpatient or two outpatient diagnoses, on different days. Consistent with retrospective evaluation of warfarin and dabigatran (2,4) |
| Stroke or Transient Ischemic Attack (TIA) | 362.3, 434.91, 435.8, 435.9, 437.1, 437.9, 438, 438.1, 438.11, 438.12, 438.13, 438.19, 438.2, 438.21, 438.22, 438.3, 438.31, 438.32, 438.4, 438.41, 438.42, 438.5, 438.51, 438.52, 438.53, 438.81, 438.82, 438.84, 438.85, 438.89, 438.9, 431, 432, 432.1, 432.9, 430, V12.54, 853.09, 853.06, 853.05, 853.04, 853.03, 853.02, 853.01, 853, 852.29, 852.26, 852.25, 852.24, 852.23, 852.22, 852.21, 852.2, 852.09, 852.06, 852.05, 852.04, 852.03, 852.02, 852.01, 852, 433.01, 433.11, 433.21, 433.31, 433.81, 433.91, 434.01, 434.11 | In a Medicare beneficiaries who were hospitalized with atrial fibrillation identified using the national Registry of Atrial Fibrillation II dataset, 2998 to 2999 was confirmed with a current stroke/TIA or past stroke/TIA with PPV = 96% (5-6) |
| Renal Disease | 583, 583.1, 583.2, 583.4, 583.6, 583.7, 588, 585.1, 585.2, 585.3, 585.4, 586, 585.6, 593.81, 593.89, 593.9, V45.11, V42.0, 582.81 | Consistent with retrospective evaluation of warfarin and dabigatran (2) |
| Pulmonary Disease | V12.60, V12.61, V12.69, 417.9, 417.8, 417, 416.9, 416.8, 416.2, 453.86, 453.87, 514, 415, 415.11, 415.12, 415.13, 415.19, 518.4, 582.89, V42.6 |  |
| Peripheral Arterial Disease | 451, 451.11, 451.19, 451.2, 451.81, 451.82, 451.83, 451.84, 451.89, 451.9, 444.01, 444.09, 444.1, 444.21, 444.22, 444.81, 444.89, 444.9, 453.1, 453.2, 453.3, 453.4, 453.41, 453.42, 453.5, 453.51, 453.52, 453.6, 453.71, 453.72, 453.73, 453.74, 453.75, 453.76, 453.77, 453.79, 453.81, 453.82, 453.83, 453.84, 453.85, 453.89, 453.9, 459.1, 459.11, 459.12, 459.13, 459.19, 459.2, 459.3, 459.31, 459.32 | Consistent with retrospective evaluation of warfarin and dabigatran (2) |
| Myocardial Infarction or Coronary Artery Disease | 413.9, 413.1, 413, 411.1, 411, 396.9, 396.8, 396.3, 396.2, 396.1, 396, 394.9, 394.2, 394.1, 394, 412, 414.2, 414.3, 414.4, 414.8, 414.9, 435, 435.1, 435.2, 435.3, 410, 410.01, 410.02, 410.1, 410.11, 410.12, 410.2, 410.21, 410.22, 410.3, 410.31, 410.32, 410.4, 410.41, 410.42, 410.5, 410.51, 410.52, 410.6, 410.61, 410.62, 410.7, 410.71, 410.72, 410.8, 410.81, 410.82, 410.9, 410.91, 410.92, 459.89, 459.9, 443.21, 443.22, 443.23, 443.24, 443.29, 440, 440.1, 440.2, 440.21, 440.22, 440.23, 440.24, 440.29, 440.3, 440.31, 440.32, 440.4, 440.8, 440.9, 414, 414.01, 414.02, 414.03, 414.04, 414.05, 414.06, 414.07, 433, 433.1, 433.2, 433.3, 433.8, 433.9, 434, 434.1, 434.9, 411.81 | Consistent with retrospective evaluation of warfarin and dabigatran (1,7) |
| Hypertension | 437.2, 401, 401.1, 401.9, 405.99, 405.91, 405.19, 405.11, 405.09, 405.01, 404.93, 404.92, 404.91, 404.13, 404.12, 404.11, 404.1, 404.03, 404.02, 404.01, 404, 403.91, 403.9, 403.11, 403.1, 403.01, 403, 402.91, 402.9, 402.11, 402.1, 402.01, 402, 416 | Consistent with retrospective evaluation of warfarin and dabigatran (2,4) |
| Hepatic | 572.8, 573.3, 573.9, 571, 571.1, 571.2, 571.3, 571.4, 571.41, 571.42, 571.49, 571.5, 571.6, 571.8, 571.9 | Consistent with retrospective evaluation of warfarin and dabigatran (2) |
| Diabetes | 362.07, 362.06, 362.05, 362.04, 362.03, 357.2, 362.01, 362.02, 366.41, 249, 249.01, 249.1, 249.11, 249.2, 249.21, 249.3, 249.31, 249.4, 249.41, 249.5, 249.51, 249.6, 249.61, 249.7, 249.71, 249.8, 249.81, 249.9, 249.91, 250, 250.01, 250.02, 250.03, 250.1, 250.11, 250.12, 250.13, 250.2, 250.21, 250.22, 250.23, 250.3, 250.31, 250.32, 250.33, 250.4, 250.41, 250.42, 250.43, 250.5, 250.51, 250.52, 250.53, 250.6, 250.61, 250.62, 250.63, 250.7, 250.71, 250.72, 250.73, 250.8, 250.81, 250.82, 250.83, 250.9, 250.91, 250.92, 250.93 | Consistent with retrospective evaluation of warfarin and dabigatran (2) |
| Chronic Heart Failure | 398.91, 428, 428.1, 428.2, 428.21, 428.22, 428.23, 428.3, 428.31, 428.32, 428.33, 428.4, 428.41, 428.42, 428.43, 428.9, 425.9, 425.8, 425.7, 425.5, 425.4, 425.18, 425.11, 411.89 | Validated in Medicare algorithm: Hospital discharge ICD-9 codes (5,8) |

**REFERENCES**

1. Graham DJ, Reichman ME, Wernecke M, Zhang R, Southworth MR, Levenson M, et al. Cardiovascular, bleeding, and mortality risks in elderly Medicare patients treated with dabigatran or warfarin for nonvalvular atrial fibrillation. Circulation. 2015;131:157-64.

2. Lauffenburger JC, Farley JF, Gehi AK, Rhoney DH, Brookhart MA, Fang G. Effectiveness and safety of dabigatran and warfarin in real-world US patients with non-valvular atrial fibrillation: a retrospective cohort study. J Am Heart Assoc. 2015;4.

3. Hernandez I, Zhang Y. Risk of Bleeding With Dabigatran in 2010-2011 Medicare Data. JAMA Intern Med. 2015;175:1245-7.

4. Rothendler JA, Rose AJ, Reisman JI, Berlowitz DR, Kazis LE. Choices in the use of ICD-9 codes to identify stroke risk factors can affect the apparent population-level risk factor prevalence and distribution of CHADS2 scores. Am J Cardiovasc Dis. 2012;2:184-91.

5. Birman-Deych E, Radford MJ, Nilasena DS, Gage BF. Use and effectiveness of warfarin in Medicare beneficiaries with atrial fibrillation. Stroke. 2006;37:1070-4.

6. Andrade SE, Harrold LR, Tjia J, Cutrona SL, Saczynski JS, Dodd KS, et al. A systematic review of validated methods for identifying cerebrovascular accident or transient ischemic attack using administrative data. Pharmacoepidemiol Drug Saf. 2012;21 Suppl 1:100-28.

7. Lauffenburger JC, Rhoney DH, Farley JF, Gehi AK, Fang G. Predictors of gastrointestinal bleeding among patients with atrial fibrillation after initiating dabigatran therapy. Pharmacotherapy. 2015;35:560-8.

8. Birman-Deych E, Waterman AD, Yan Y, Nilasena DS, Radford MJ, Gage BF. Accuracy of ICD-9-CM codes for identifying cardiovascular and stroke risk factors. Med Care. 2005;43:480-5.

9. Lin KJ, Singer DE, Glynn RJ, Blackley S, Zhou L, Liu J, et al. Prediction Score for Anticoagulation Control Quality Among Older Adults. J Am Heart Assoc. 2017;6.

10. Yu AY, Malo S, Wilton S, Parkash R, Svenson LW, Hill MD. Anticoagulation and population risk of stroke and death in incident atrial fibrillation: a population-based cohort study. CMAJ Open. 2016;4:E1-6.

11. Roumie CL, Mitchel E, Gideon PS, Varas-Lorenzo C, Castellsague J, Griffin MR. Validation of ICD-9 codes with a high positive predictive value for incident strokes resulting in hospitalization using Medicaid health data. Pharmacoepidemiol Drug Saf. 2008;17:20-6.

12. Cunningham A, Stein CM, Chung CP, Daugherty JR, Smalley WE, Ray WA. An automated database case definition for serious bleeding related to oral anticoagulant use. Pharmacoepidemiol Drug Saf. 2011;20:560-6.

13. Wahl PM, Rodgers K, Schneeweiss S, Gage BF, Butler J, Wilmer C, et al. Validation of claims-based diagnostic and procedure codes for cardiovascular and gastrointestinal serious adverse events in a commercially-insured population. Pharmacoepidemiol Drug Saf. 2010;19:596-603.
