# Supplementary material for: Evaluating the effects of socioeconomic status on stroke and bleeding risk scores and clinical events in patients on oral anticoagulant for new onset atrial fibrillation
Source: PLoS One. 2021 Mar 18;16(3):e0248134. doi: 10.1371/journal.pone.0248134 (PMC7971564; doi:10.1371/journal.pone.0248134)
Supplement: S2 Table — aAll clinical events were identified using inpatient diagnosis only. (DOCX) [file pone.0248134.s003.docx]

| Table S2. List of ICD-9 and ICD-10 codes used to identify clinical events^a^ | | | |
| --- | --- | --- | --- |
| Event | ICD-9 Codes | Comment | ICD-10 Codes |
| Systematic Emboli | 362.3, 444.01, 444.09, 444.1, 444.89, 444.9, 451.89, 451.9, 453.1, 453.2, 453.86, 453.87, 453.89, 453.9 | Consistent with a recent SAMe-TT_2_R2 evaluation in patients on warfarin with AF and history of venous thrombus embolism (9) | H34.9, I74.01, I74.09, I74.10, I74.11, I74.19, I74.8, I74.9, I80.8, I80.9, I82.210, I82.220, I82.221, I82.290, I82.890, I82.891, I82.90, I82.91 |
| Stroke and TIA | 362.3, 430, 431, 433.01, 433.11, 433.21, 433.31, 433.81, 433.91, 434.01, 434.11, 434.91, 435.8, 435.9, 436 | In Tennessee Medicaid enrollees aged 50-84, the algorithm had PPV of 97% for primary discharge (10),  Consistent with prior OAC population and stroke studies (11) and Consistent with retrospective evaluation of warfarin and dabigatran (1,12)  See Mini-Sentinel report for PPV for individual codes or other algorithms (11) | G45.1, G45.2, G45.8, G45.9, G46.0, G46.1, G46.2, G46.3, G46.4, G46.5, G46.6, G46.7, H34.9, I63.00, I63.011, I63.012, I63.019, I63.02, I63.031, I63.032, I63.039, I63.09, I63.10, I63.111, I63.112, I63.119, I63.12, I63.131, I63.132, I63.139, I63.19, I63.20, I63.211, I63.212, I63.219, I63.22, I63.231, I63.232, I63.239, I63.29, I63.30, I63.311, I63.312, I63.319, I63.321, I63.322, I63.329, I63.331, I63.332, I63.339, I63.341, I63.342, I63.349, I63.39, I63.40, I63.411, I63.412, I63.419, I63.421, I63.422, I63.429, I63.431, I63.432, I63.439, I63.441, I63.442, I63.449, I63.49, I63.50, I63.511, I63.512, I63.519, I63.521, I63.522, I63.529, I63.531, I63.532, I63.539, I63.541, I63.542, I63.549, I63.59, I63.6, I63.8, I63.9, I67.81, I67.82 |
| Intracranial Bleeding | 430, 431, 432, 432.1, 432.9 | Consistent with retrospective evaluation of warfarin and dabigatran (1,12) | I60.00, I60.01, I60.02, I60.10, I60.11, I60.12, I60.20, I60.21, I60.22, I60.30, I60.31, I60.32, I60.4, I60.50, I60.51, I60.52, I60.6, I60.7, I60.8, I60.9, I61.0, I61.1, I61.2, I61.3, I61.4, I61.5, I61.6, I61.8, I61.9, I62.00, I62.01, I62.02, I62.03, I62.9 |
| GI Bleeding | 456, 456.2, 530.82, 531, 531.01, 531.2, 531.21, 531.4, 531.41, 531.6, 531.61, 532, 532.01, 532.2, 532.21, 533, 533.01, 533.2, 533.21, 533.4, 534, 534.01, 534.2, 534.21, 535.01, 535.11, 535.21, 535.31, 535.41, 535.51, 535.61, 535.71, 537.83, 562.02, 562.03, 562.12, 562.13, 569.3, 569.85, 578.1, 578.9 | Consistent with retrospective evaluation of warfarin and dabigatran (1-2,12)  PPVs for individual codes (12-13) | I85.01, I85.11, K25.0, K25.2, K25.4, K25.6, K26.0, K26.2, K27.0, K27.2, K27.4, K28.0, K28.2, K29.01, K29.21, K29.31, K29.41, K29.51, K29.61, K29.71, K29.81, K29.91, K31.811, K55.21, K57.01, K57.11, K57.13, K57.21, K57.31, K57.33, K57.41, K57.51, K57.53, K57.81, K57.91, K57.93, K62.5, K92.1, K92.2 |
| Other Major Bleeding | 423, 459, 568.81, 578, 599.7, 623.8, 719.1, 784.7, 784.8, 786.3, 786.3 | Consistent with retrospective evaluation of warfarin and dabigatran (1)  PPVs for individual codes (12-13) | I31.2, K66.1, K92.0, R04.0, R04.1, R04.2, R04.9, R58 |

^a^All clinical events were identified using inpatient diagnosis only.
