# Supplementary material for: Evaluating the effects of socioeconomic status on stroke and bleeding risk scores and clinical events in patients on oral anticoagulant for new onset atrial fibrillation
Source: PLoS One. 2021 Mar 18;16(3):e0248134. doi: 10.1371/journal.pone.0248134 (PMC7971564; doi:10.1371/journal.pone.0248134)
Supplement: S3 Table — Variables included ADI with a threshold of 60%, as well as TTR thresholds of 40% and 65% in the first year of therapy. Hazard ratios in bold indicate statistical significance (p<0.05). Ischemic events include Systemic Embolic Events and Stroke TIA. Bleeding events includes, GI-bleeding, non-GI bleeding and intracranial hemorrhage. (DOCX) [file pone.0248134.s004.docx]

| **Table S3:** Multivariate analysis using cox regression to predict ischemic and bleeding events in patients with a previous history of stroke prior to initiating warfarin (secondary stroke prevention). Variables included ADI with a threshold of 60%, as well as TTR thresholds of 40% and 65% in the first year of therapy. | | | |
| --- | --- | --- | --- |
|  | **High-ADI**  **ADI>60%** | **TTR <40%** | **TTR >65%** |
|  | *Hazard Ratio (CI)* | *Hazard Ratio (CI)* | *Hazard Ratio (CI* |
| **Ischemic** | 1.51 (0.95-2.39) | **2.43 (1.39-4.26)** | 1.47 (0.82-2.63) |
| **Bleeding** | 0.57 (0.19-1.63) | 1.26 (0.51-3.12) | 0.86 (0.34-2.12) |
| **Death** | 0.92 (0.40-2.13) | **3.79 (1.61-8.89)** | 0.45 (0.13-1.56) |
| Hazard ratios in bold indicate statistical significance (p<0.05). Ischemic events include Systemic Embolic Events and Stroke TIA. Bleeding events includes, GI-bleeding, non-GI bleeding and intracranial hemorrhage. | | | |
